# Long‐term outcomes after cytoreductive partial nephrectomy for metastatic renal cell carcinoma

**DOI:** 10.1002/bco2.70122

**Published:** 2026-01-12

**Authors:** Andrea Lopez Sanmiguel, Yash S. Khandwala, Emily A. Vertosick, Daniel Barbakoff, Roya Ghavamian, Jonathan A. Coleman, Mark Dawidek, Andrew J. Vickers, A. Ari Hakimi, Paul Russo

**Affiliations:** ^1^ Urology Service, Department of Surgery Memorial Sloan Kettering Cancer Center New York New York USA; ^2^ Department of Epidemiology and Biostatistics Memorial Sloan Kettering Cancer Center New York New York USA

**Keywords:** cytoreductive partial nephrectomy, metastatic renal cell carcinoma, patient selection, renal function, survival analysis

## Abstract

**Objectives:**

To assess treatment outcomes and evaluate patient selection criteria for cytoreductive partial nephrectomy (CRPN) in a unique cohort of metastatic renal cell carcinoma (mRCC) patients.

**Methods:**

A retrospective review of mRCC patients who underwent CRPN between 1995 and 2023 at a single institution was performed. Clinical characteristics, perioperative outcomes, longitudinal imaging reports and overall survival data were analysed.

**Results:**

Seventy‐three patients with mRCC were included. Forty per cent of patients had prior radical nephrectomy, and 44% had prior metastasectomy. The median tumour size was 4 cm (IQR 2.7, 5.5). Median follow‐up among patients who survived was 6.7 years (IQR 3.4, 9.6). Median overall survival was 6.1 years (95% CI 4.6 to 7.8). Complications occurred in 22% of patients within 30 days post‐surgery. eGFR stabilized at 3 months after surgery, and no patients required dialysis. Larger tumour size was associated with a higher risk of cancer‐specific death (HR 1.19, 95% CI 1.07 to 1.31, *p* < 0.001). Higher pathologic stage and grade were associated with significantly higher risks of cancer‐specific death (HR 2.78, 95% CI 0.83 to 9.36, *p* = 0.10 and HR 1.45, 95% CI 0.64 to 3.29, *p* = 0.4, respectively).

**Conclusion:**

CRPN was performed effectively as a component of integrated medical and surgical management for highly selected mRCC patients. Preservation of renal function in patients with a solitary kidney or with an intact contralateral kidney was achieved with acceptable surgical morbidity and oncologic outcomes.

## 1 INTRODUCTION

With the advent of contemporary systemic therapy combinations for metastatic renal cell carcinoma (mRCC), the role of cytoreductive nephrectomy (CRN) is becoming increasingly pertinent. CRN is highly dependent on careful case selection, and clinical trials such as CARMENA indicate its benefits are likely restricted to patients with good‐ or intermediate‐risk disease.^1^, ^2^ Though, even for poor‐risk patients, a strong radiographic response combined with favourable performance status may allow for added benefit from delayed or ‘integrated cytoreduction’.^3^

As a result, CRN continues to have a role in the management of mRCC, and ongoing prospective randomized clinical trials may further elucidate its place within the current treatment landscape.^3^, ^4^, ^5^ The role of CRN may even continue to grow as survival times improve due to optimized immunotherapy combinations. Recent analyses from the CheckMate 214 trial demonstrated strong objective response rates for nivolumab (anti‐PD‐1) and ipilimumab (anti‐CTLA‐4), ranging from 41% to 55%, with complete response rates of 9% in intermediate‐ and poor‐risk patients with advanced RCC.^6^, ^7^

Given these high response rates and improved survival, quality of life and long‐term toxicity considerations are becoming more relevant. For select patients with mRCC who are candidates for CRN, cytoreductive partial nephrectomy (CRPN) may be considered if they have a solitary kidney, pre‐existing chronic kidney disease, or a small easily accessible tumour.^6^, ^7^, ^8^

Although some retrospective studies have shown non‐inferior cancer‐specific outcomes when comparing partial with radical CRN, these studies have been limited by small cohort sizes, a lack of contemporary real‐world data and have yet to be evaluated in a randomized clinical trial.^9^, ^10^ Clearly, only a select group of patients is expected to benefit from a nephron‐sparing approach in the setting of metastatic disease, and there remains a need to better identify these patients.^1^

Our study aims to evaluate the clinical impact of CRPN in mRCC patients, focusing on assessing its feasibility, safety and long‐term outcomes. We additionally sought to identify key differences in clinical characteristics between patients undergoing CRPN and cytoreductive radical nephrectomy (CRRN) at our institution to highlight important factors in patient selection criteria.

## 2 METHODS

Using our prospectively maintained kidney tumour database, we identified a cohort of mRCC patients aged ≥18 years who underwent CRPN at our institution between 1995 and 2023. Patients were excluded from the study if the nephrectomy was not performed for a primary renal tumour. A retrospective review of electronic medical records (EMRs) was subsequently conducted for all study patients to extract clinical characteristics, perioperative outcomes, longitudinal imaging reports, and overall survival (OS) data. Cause of death accounted for whether a patient died from mRCC, from another cancer type or unrelated cause based on EMRs. To compare selection criteria, a cohort of mRCC patients aged ≥18 years who underwent CRRN during the same period was also identified and described. Survival comparisons between the two cohorts were not performed as large baseline differences between the groups were expected. Manual chart review of preoperative notes was performed to identify the indications for CRPN.

## 3 STATISTICAL ANALYSIS

To assess differences in clinical characteristics between patients undergoing CRPN and CRRN, patient and tumour characteristics were compared using Wilcoxon rank sum and Fisher's exact tests.

All other analyses were limited to CRPN patients only. Postoperative complications were assessed within 30 days following surgery and classified according to the Clavien–Dindo classification.^11^ For patients that experienced multiple complications, each event was recorded and counted separately. Local recurrence after CRPN was defined as the need for a subsequent ipsilateral nephrectomy or the detection of a new lesion within the area of the CRPN on imaging. Kaplan–Meier curves were generated to evaluate cancer‐specific survival (CSS) and OS.

To evaluate estimated glomerular filtration rate (eGFR) after CRPN, we calculated eGFR and chronic kidney disease (CKD) stage at 3 months (±1 month) and 12 months (±2 months) separately for patients with and without prior radical nephrectomy (RN). A generalized additive model was used to generate a graph depicting mean eGFR over time during the first 15 months after surgery, comparing patients with and without prior RN.

A Cox regression model was developed to identify predictors of cancer‐specific death. Due to the small cohort size and low number of events, we were limited to a univariable model. The following predictors of interest were assessed: age at nephrectomy, sex, preoperative eGFR, prior RN, prior metastasectomy, tumour size on pathology, pathologic stage (T3 and T4 vs. T1 and T2) and grade (high vs. low), histology (clear cell vs. non‐clear cell), positive surgical margins, number and site of metastases before partial nephrectomy (0 vs. 1 and 2 vs. ≥3) and size of the largest metastasis before CRPN. For the analysis of the size of the largest metastasis before partial nephrectomy, patients without metastatic disease at the time of surgery were excluded. All analyses were conducted using R version 4.4.0 with the gtsummary (v1.7.2) and tidyverse (v2.0.0) packages.^12^, ^13^, ^14^

## 4 RESULTS

A total of 80 mRCC patients scheduled for CRPN met the inclusion criteria. After an initial chart review, seven patients were excluded due to surgery for a non‐primary renal neoplasm. As a result, 73 patients with mRCC remained in the final analysis. Among these, four cases required conversion to RN during surgery; because their intended procedure was a partial nephrectomy, they were included in the analysis on an intent‐to‐treat basis.

Patient and disease characteristics are provided in Table 1. The CRPN cohort consists of mostly favourable‐ to intermediate‐risk mRCC patients, with 92% (*N* = 34) of those with available IMDC scores categorized as such. The majority were male (75%) with a median age of 63 years. A significant proportion had undergone prior RN (40%, *N* = 29) and metastasectomy (44%, *N* = 32). These characteristics reflect a highly selected and heterogeneous cohort.

**TABLE 1:** Patient and disease characteristics for cytoreductive partial nephrectomy and cytoreductive radical nephrectomy.

Characteristic	Partial *N* = 73	Radical *N* = 564	*p* value
Male	55 (75%)	410 (73%)	0.6
BMI	28 (25, 31)	28 (25, 32)	>0.9
Unknown	11	131	
Age	63 (56, 71)	61 (53, 68)	0.073
Smoking history			>0.9
Current	10 (14%)	81 (15%)	
Former	29 (40%)	212 (40%)	
Never	34 (47%)	235 (45%)	
Unknown	0	36	
Diabetes	9 (12%)	88 (16%)	0.5
Coronary artery disease	5 (6.8%)	45 (8.0%)	0.7
Preoperative eGFR	67 (57, 84)	79 (63, 95)	<0.001
Unknown	0	1	
Preoperative calcium	9.3 (9.0, 9.6)	9.5 (9.1, 9.9)	0.003
Unknown	3	46	
Preoperative LDH	177 (157, 197)	182 (155, 218)	0.3
Unknown	21	253	
Preoperative haemoglobin	13.3 (12.3, 14.3)	12.4 (10.8, 13.9)	<0.001
Unknown	3	36	
Preoperative systemic treatment	18 (25%)	85 (15%)	0.036
Approach			0.038
Open	50 (68%)	456 (81%)	
Laparoscopic	7 (9.6%)	40 (7.1%)	
Robotic	16 (22%)	68 (12%)	
Pathologic T stage			<0.001
≤T1	41 (59%)	53 (9.5%)	
T2	4 (5.7%)	45 (8.0%)	
≥T3	25 (36%)	462 (83%)	
Unknown	3	4	
Pathologic high grade	53 (78%)	446 (91%)	0.002
Unknown	5	72	
Tumour size at pathology (cm)	4.0 (2.7, 5.5)	8.7 (6.4, 11.3)	<0.001
Unknown	0	6	
Tumour histology			0.4
Clear cell	58 (79%)	450 (81%)	
Papillary	6 (8.2%)	17 (3%)	
Other	9 (13%)	91 (16%)	
Unknown	0	6	
Positive surgical margins	10 (14%)	69 (13%)	0.8
Unknown	0	15	
Length of stay, in days	3 (2, 6)	3 (2, 5)	0.5
Unknown	2	42	

*Note*: Data are presented as median (IQR) and *N* (%).

The CRRN cohort used for comparison included 564 patients. A comparison of baseline characteristics between the two groups (Table 1) found statistically significant differences in baseline haemoglobin, calcium and eGFR, tumour size, pathologic T stage and grade. The CRPN cohort had a smaller median tumour size (4.0 cm vs. 8.7 cm, *p* < 0.001), as well as lower pathologic T stage (*p* < 0.001). Eighteen patients (25%) in the CRPN cohort received prior systemic therapy compared with 85 (15%) in the CRRN group (*p* = 0.036). No statistically significant differences were observed in age, sex, diabetes, coronary artery disease or histology type.

There were no further metastatic progression events between the time of prior treatment and CRPN. Table 2 outlines the location of metastases at diagnosis and at the time of CRPN. The most common site of initial metastatic disease was the lung, followed by bone and retroperitoneal lymph nodes. Some patients underwent metastasectomy or received other treatment strategies, including systemic therapy and ablative techniques, which altered their metastatic pattern.

**TABLE 2:** Location of metastases at diagnosis and location, number and size of metastasis at the time of cytoreductive partial nephrectomy.

Characteristic	At diagnosis	Pre‐nephrectomy
	*N* = 73	*N* = 73
Adrenal	8 (11%)	5 (8.8%)
Bone	19 (26%)	11 (19%)
Liver	5 (6.8%)	4 (7.0%)
Lymph nodes	18 (25%)	16 (28%)
Lung	35 (48%)	33 (58%)
Other	10 (14%)	1 (1.8%)
Pancreas	6 (8.2%)	4 (7.0%)
None		16 (22%)
Number of metastases before index surgery		
0		16 (22%)
1		12 (16%)
2		11 (15%)
3		10 (14%)
4		6 (8.2%)
≥5		18 (25%)
Largest metastasis before index surgery (cm)		2.1 (1.3, 3.3)

*Note*: Some patients had no metastases at the time of surgery due to receiving metastasectomy or systemic treatment. Percentages may sum to >100% as patients could have multiple metastasis sites. Data are presented as median (IQR) and *N* (%).

Eighteen patients (25%) received preoperative systemic therapy; the most used agent was sunitinib (six patients, 33%), followed by immune check point inhibitors (ICIs) (four patients, 22%), including nivolumab, ipilimumab and pembrolizumab. Postoperatively, seven patients (9.6%) received systemic therapy within the first 3 months following surgery, including three patients treated with TKIs (43%) and two with ICIs (29%), one received nivolumab and the other received ipilimumab. A total of 17 patients (23%) received systemic therapy 3 months after surgery, with TKIs being the most commonly used agent in seven of them (41%). Detailed treatment data are presented in Table S1.

**FIGURE 1:**
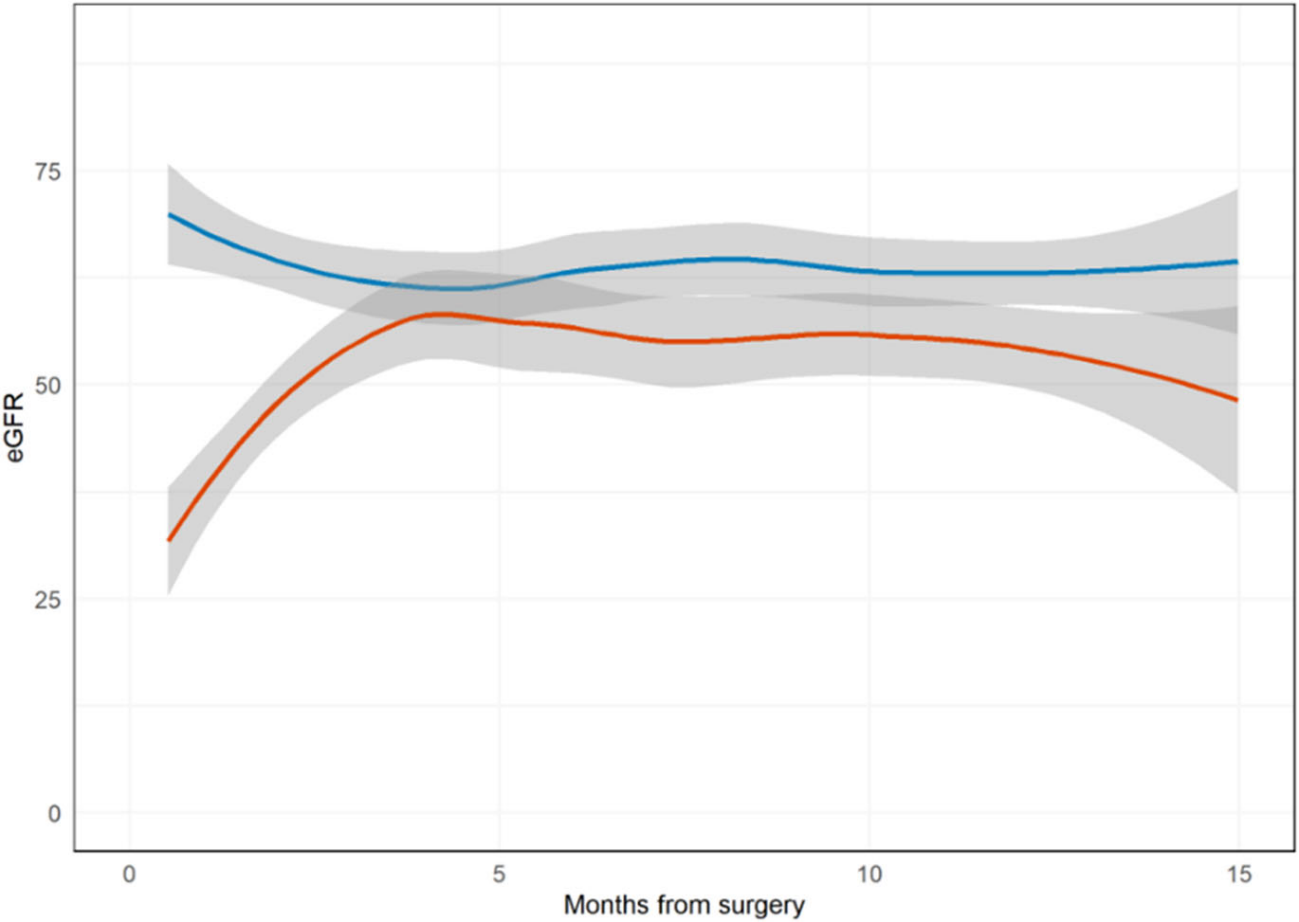
eGFR over time for the first 15 months after CRPN, for those who underwent a prior radical nephrectomy (red line) and those who did not (blue line).

Figure 1 illustrates the eGFR trend over the first 15 months post‐surgery stratified by prior RN status, including data from 70 patients with at least one eGFR measurement before 15 months. eGFR was lower in patients with a prior RN, with eGFR levels in both groups stabilizing around 3 months. Table S2 presents the eGFR and CKD stage at 3 and 12 months for these two groups. Within 30 days after surgery, 16 patients (22%) experienced a total of 27 complications, as some patients had more than one complication. These included 14 Clavien–Dindo grade I/II and 13 grade III complications (Table 3).

**TABLE 3:** Postoperative complications within 30 days following surgery.

Type of complication	*N*
Clavien I/II	
Renal	3
Respiratory	3
Gastrointestinal	1
Other	7
Clavien III	
Renal	8
Respiratory	2
Other	3
Total	27

*Note*: Patients may be listed more than once if they had multiple complications.

**FIGURE 2:**
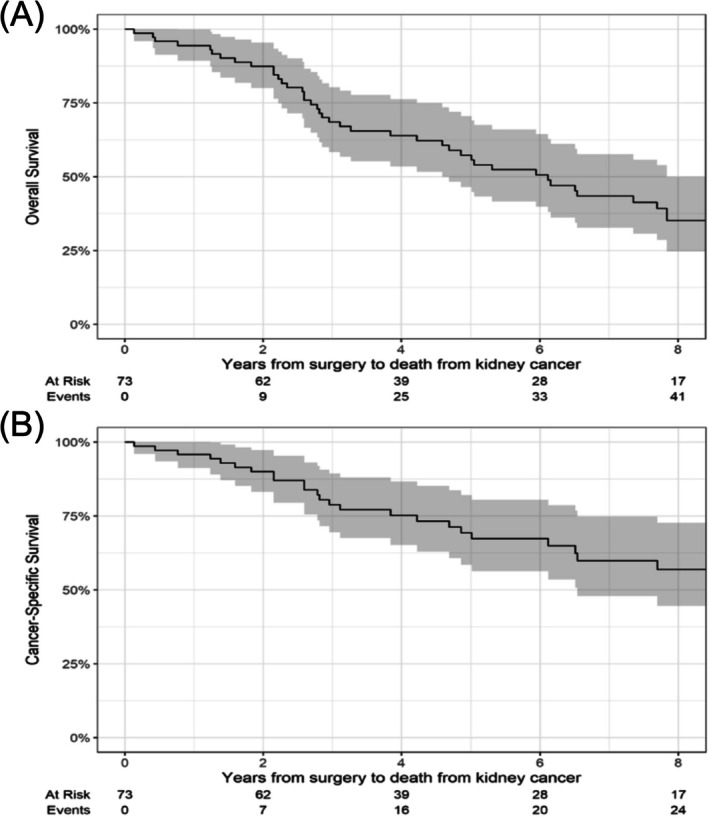
Kaplan–Meier curves for the outcomes of time to death from RCC (A) and death from any cause (B).

A total of 25 (34%) patients died from mRCC during the study period. The median follow‐up among patients who survived was 6.7 years (IQR 3.4, 9.6). Kaplan–Meier curves for CSS and OS are presented in Figure 2. The median OS was 6.1 years (95% CI 4.6 to 7.8). The median CSS was not reached. Of note, during the time frame of this study, the CSS was shifting due to improved effectiveness in available systemic agents.

We then investigated patient and disease characteristics associated with cancer‐specific death; all variables assessed are presented in Table S3. There was evidence that male patients are at greater risk of cancer‐specific death (HR 3.78, 95% CI 0.89 to 16.1, *p* = 0.072), although this association did not reach statistical significance, and the confidence interval was wide. Larger tumour size on pathology was related to an increased risk of cancer‐specific death (HR 1.19 per 1 cm increase, 95% CI 1.07 to 1.31, *p* < 0.001). Additionally, an increasing number of metastases at the time of partial nephrectomy, particularly with ≥3, was associated with a higher risk of cancer‐specific death (HR 2.42, 95% CI 0.87 to 6.76, *p* = 0.091).

Several key factors influenced a surgeon's decision to perform CRPN over CRRN. The most common reason was a history of prior RN in 29 patients (40%), followed by tumour location, particularly the presence of an exophytic mass in 16 cases (22%), and stable disease following metastasectomy or systemic therapy in 15 patients (21%). Less frequently, CRPN was performed in five patients (7%) with bilateral renal masses, three patients (4%) with chronic kidney disease and four (5%) with multiple comorbidities.

## 5 DISCUSSION

This study presents long‐term outcomes from a single institutional experience with CRPN for mRCC in a carefully selected group of 73 patients. Forty per cent had a solitary kidney after previous contralateral RN for RCC and 44% had prior RCC metastasectomy. CRPN was found to effectively preserve renal function. This quality‐of‐life benefit is relevant in the setting of longer OS times for mRCC patients currently achieved by modern immunotherapy combinations.^8^, ^15^, ^16^ By comparing baseline characteristics between the CRPN and CRRN cohorts, we identified factors guiding patient selection. Essential indications included a solitary kidney and reduced eGFR in the setting of CKD, while elective indications were for small exophytic tumours and stable disease following metastasectomy or systemic therapies.

While CRRN remains more common, CRPN accounted for 11% of all cytoreductive procedures at our high‐volume cancer centre over a 28‐year timeframe (1995–2023). This rate is higher than what other studies have reported, but it aligns with the increased use observed in recent studies, including a retrospective analysis of the SEER database by Lenis et al., which documented a rise in CRPN between 2006 and 2013 with an overall rate of 3.8%.^6^, ^7^, ^17^

Our cohort consisted predominantly of patients with small primary tumours, consistent with patterns seen in prior CRPN studies, and a manifestation of strict case selection. Hauser et al. used the National Cancer Database and found that 54% of patients treated with CRPN had T1 stage tumours, despite T1 tumours representing only 17% of all cytoreductive cases.^10^ Mazzone et al. further reported that 32% of patients in the CRPN group had grade 1 and 2 tumours, compared with 20% in the CRRN group.^10^, ^18^ Although our cohort had a slightly higher proportion of high‐grade tumours, the overall distribution of stage and grade remained comparable with prior cohorts.

Additionally, our CRPN cohort had lower baseline eGFR values compared with patients undergoing CRRN. Babaian et al. reported a median preoperative eGFR of 55 mL/min, with a postoperative value of 49 mL/min, which is modestly lower than our cohort's median preoperative eGFR of 67 mL/min. However, comparisons of perioperative renal function are limited by a lack of long‐term eGFR data published in prior studies.^19^, ^20^ In our cohort, patients experienced an initial drop in eGFR following surgery, which stabilized by 3 months, with no significant changes at 12 months post‐surgery. Importantly, none of the patients required dialysis.

A concern with CRPN is its technical complexity, which has been associated with increased complication rates.^10^, ^19^, ^21^ In our study, the 30‐day postoperative complication rate following CRPN was 22%. Similarly, Krambeck et al. reported complication rates of 22% to 33% for CRPN, compared with 9.9% in the CRRN group.^21^ The majority of complications in our study were Clavien–Dindo grade III and primarily kidney related, with urinary leak or fistula being the most common. This pattern is consistent with Babian et. al who also reported urinary leak as the most frequent complication.^19^

The rate of positive surgical margins in our study was comparable with the CRRN cohort (14% vs. 13%). While not reaching conventional levels of statistical significance, we did find evidence of a clinically relevant association between positive surgical margins and an increased hazard of cancer‐specific death. Studies using larger datasets, including Lenis et al. have observed a higher positive margin rate in CRPN (22%) but reported a similar effect size indicating worse OS for patients with positive margins (HR 1.47, 95% CI 1.39 to 1.55).^10^, ^17^ However, their analysis included both CRPN and CRRN patients and did not assess CSS.^17^ Other previous single‐centre studies did not report on positive surgical margin rates.^19^, ^21^, ^22^

Our findings show that CRPN has an OS of 6.1 years (95% CI 4.6 to 7.9). However, it is important to note that this is a highly selected and heterogeneous cohort, and its favourable characteristics likely contributed to these observed outcomes. This aligns with the findings of Garcia et al., who analysed the SEER database and found that CRPN was an independent predictor of lower overall mortality compared with CRRN. However, despite all being mRCC patients, the CRPN group had smaller tumours and more favourable grades making causal inference difficult.^9^

In contrast, a separate SEER‐based study focusing on T1 and T2 M1 RCC cases found no significant difference in OS or CSS between the CRPN and CRRN groups, despite the CRRN group having larger tumours.^6^ Furthermore, Singla et al. reported that immunotherapy administered prior to CRN was associated with a lower pathologic T stage, smaller tumour size and superior OS.^8^ This reinforces the potential advantages of integrating immunotherapy with CRPN in the treatment of mRCC, particularly for patients with T1 and T2 tumours.

At baseline, the lung was the most common metastatic site in this cohort, which indicates a more favourable disease pattern.^23^ Additionally, most patients had low‐volume metastatic disease, and previous studies including Tian et al., identified ≥2 metastases as the most prominent risk factor for both OS and CSS. Furthermore, Chen et al. reported better survival outcomes in patients with isolated metastasis treated with CRPN.^6^, ^24^ In terms of cancer‐specific mortality, tumour size was the only statistically significant predictor, which aligns with the existing literature suggesting that a primary tumour size ≤4 cm is associated with improved OS in mRCC.^17^, ^19^, ^24^, ^25^

Although our study provides valuable insights, there are several important limitations. This is a retrospective study of an infrequent treatment strategy for mRCC. Additionally, data were accumulated over a 28‐year period during which the partial nephrectomy technique has evolved substantially and contemporary systemic treatment for mRCC has changed. These cases were performed at a high‐volume cancer centre with substantial surgical expertise and multidisciplinary support, and thus, outcomes may not be applicable to smaller, community settings. Nevertheless, this study suggests the feasibility and favourable outcomes of CRPN in a select group of patients.

## 6 CONCLUSION

CRPN in mRCC is a safe and effective approach that preserves renal function. Survival was long in this highly selected subgroup of patients undergoing CRPN. Patient selection is key, with small, favourably located tumours and solitary kidneys being indications. While a prospective comparison between CRPN and CRRN would be logistically challenging, CRPN should continue to be considered in appropriate cases. Ongoing research through real‐world data and registry‐based studies will be essential to refine patient selection and further elucidate the long‐term impact of CRPN on cancer control and patient outcomes.

## AUTHOR CONTRIBUTIONS

**Andrea Lopez Sanmiguel:** Project writing; research; development. **Yash S. Khandwala:** Project writing; research; development. **Emily A. Vertosick:** Statistical analysis; editotrial comment. **Daniel Barbakoff:** Project writing; research; development. **Roya Ghavamian:** Project writing; research; development. **Jonathan A. Coleman:** Critical comments. **Mark Dawidek:** Critical comments. **Andrew J. Vickers:** Stasticial supervision. **A. Ari Hakimi:** Project writing; research; development. **Paul Russo:** Project writing; research; development.

## CONFLICT OF INTEREST STATEMENT

The authors declare no conflicts of interest related to this study.

## Supporting information


**Table S1.** Systemic therapy agents used. Data are presented as N (%).
**Table S2.** eGFR and CKD stage at 3 months and 12 months after surgery, separately for those who received a prior radical nephrectomy and those who did not. Data are presented as median (IQR) and N (%).
**Table S3.** Results of univariable Cox models for cancer‐specific death.
