# Supplementary material for: Long‐term outcomes after cytoreductive partial nephrectomy for metastatic renal cell carcinoma
Source: BJUI Compass. 2026 Jan 12;7(1):e70122. doi: 10.1002/bco2.70122 (PMC12795982; doi:10.1002/bco2.70122)
Supplement: Supplementary file 1 — Table S1. Systemic therapy agents used. Data are presented as N (%). Table S2. eGFR and CKD stage at 3 months and 12 months after surgery, separately for those who received a prior radical nephrectomy and those who did not. Data are presented as median (IQR) and N (%). Table S3. Results of univariable Cox models for cancer‐specific death. [file BCO2-7-e70122-s001.docx]

**Supplementary**

**Supplementary Table 1.** Systemic therapy agents used. Data are presented as N (%).

|  | **Before CRPN** | **<3 months after surgery** | **>3 months after surgery** |
| --- | --- | --- | --- |
| **Agent** | **N = 18** | **N = 7** | **N =17** |
| TKI | 6 (33%) | 3 (43%) | 7 (41%) |
| ICI | 4 (22%) | 2 (29%) | 1 (6%) |
| mTORi | 1 (6%) | 1 (14%) | 4 (24%) |
| Clinical Trial | 7 (39%) | 1 (14%) | 5 (29%) |

*****TKI: tyrosine kinase inhibitor, ICI: immune check point inhibitor, mTORi: mTOR receptor inhibitor.

**Supplementary Table 2.** eGFR and CKD stage at 3 months and 12 months after surgery, separately for those who received a prior radical nephrectomy and those who did not. Data are presented as median (IQR) and N (%).

|  | **3 Months** | | **12 Months** | |
| --- | --- | --- | --- | --- |
| **Characteristic** | **No Prior Radical**  N = 27 | **Prior Radical**  N = 16 | **No Prior Radical**  N = 28 | **Prior Radical**  N = 19 |
| eGFR | 60 (48, 81) | 52 (38, 61) | 63 (50, 88) | 53 (42, 59) |
| CKD stage |  |  |  |  |
| <15 | 0 (0%) | 1 (6.3%) | 0 (0%) | 1 (5.3%) |
| 15-29 | 3 (11%) | 1 (6.3%) | 1 (3.6%) | 0 (0%) |
| 30-44 | 3 (11%) | 4 (25%) | 4 (14%) | 4 (21%) |
| 45-59 | 8 (30%) | 6 (38%) | 7 (25%) | 10 (53%) |
| 60-89 | 9 (33%) | 3 (19%) | 10 (36%) | 4 (21%) |
| ≥ 90 | 4 (15%) | 1 (6.3%) | 6 (21%) | 0 (0%) |

**Supplementary Table 3.** Results of univariable Cox models for cancer-specific death.

|  | **Cancer-Specific Death** | | | |
| --- | --- | --- | --- | --- |
| **Characteristic** | **N** | **HR** | **95% CI** | **p-value** |
| Age at nephrectomy, per 5 years | 73 | 0.97 | 0.80, 1.18 | 0.8 |
| Male | 73 | 3.78 | 0.89, 16.1 | 0.072 |
| Pre-operative eGFR, per 10 mL/min/1.73m2 | 73 | 0.96 | 0.78, 1.17 | 0.7 |
| Prior radical nephrectomy | 73 | 0.68 | 0.30, 1.54 | 0.4 |
| Prior metastasectomy | 73 | 0.82 | 0.37, 1.81 | 0.6 |
| Tumor size on pathology (cm) | 73 | 1.19 | 1.07, 1.31 | <0.001 |
| Pathologic tumor grade high vs low | 68 | 2.78 | 0.83, 9.36 | 0.10 |
| Pathologic stage T3/T4 vs T1/T2 | 70 | 1.45 | 0.64, 3.29 | 0.4 |
| Positive surgical margins | 73 | 2.02 | 0.75, 5.41 | 0.2 |
| Clear cell vs non-clear cell histology | 73 | 0.66 | 0.25, 1.79 | 0.4 |
| Number of metastases before index surgery | 73 |  |  |  |
| 0 |  | — | — |  |
| 1-2 |  | 0.97 | 0.28, 3.35 | >0.9 |
| ≥ 3 |  | 2.42 | 0.87, 6.76 | 0.091 |
| Largest metastasis before index surgery (cm) | 57 | 1.17 | 0.97, 1.41 | 0.11 |
